# Piplartine as an Innovative Natural Product With Dual Antiparasitic and Immunomodulatory Actions

**DOI:** 10.1155/jimr/9793044

**Published:** 2026-02-04

**Authors:** Lucas Fukui-Silva, Felipe C. R. de Souza, Camila S. Amorim, Josué de Moraes

**Affiliations:** ^1^ Research Center on Neglected Diseases, Guarulhos University, Guarulhos, São Paulo, Brazil, ung.br; ^2^ Research Center on Neglected Diseases, Scientific and Technological Institute, Brasil University, São Paulo, São Paulo, Brazil, universidadebrasil.edu.br

**Keywords:** immunomodulation, immunoproteasome, neglected tropical diseases, NF-κB, Nrf2, piperlongumine, piplartine, redox signaling

## Abstract

Neglected tropical diseases (NTDs) remain a major global health burden, disproportionately affecting low‐ and middle‐income regions and demanding innovative therapeutic strategies. This review summarizes current evidence on piplartine (piperlongumine), a naturally occurring amide from *Piper* species with broad antiparasitic and immunomodulatory properties. Studies on *Schistosoma mansoni*, *Angiostrongylus cantonensis*, *Leishmania* spp., *Trypanosoma cruzi*, *Trypanosoma brucei*, *Plasmodium falciparum*, and *Toxoplasma gondii* demonstrate potent activity at micromolar levels, associated with disruption of parasite redox homeostasis. In host immune cells, piplartine modulates NF‐κB and Nrf2 signaling, inhibits the immunoproteasome, and promotes antioxidant and anti‐inflammatory responses. These dual properties suggest that piplartine may influence both parasite survival and host immunopathology through redox‐sensitive mechanisms, although direct cause–effect relationships during parasitic infection remain to be experimentally established. The compound’s favorable drug‐likeness, oral bioavailability, and selective toxicity profile highlight its potential as a multitarget scaffold for the development of next‐generation therapeutics against neglected tropical and immune‐related diseases.

## 1. Introduction

Neglected tropical diseases (NTDs) comprise a heterogeneous group of infectious and toxin‐related conditions caused primarily by parasites (helminths and protozoa) but also by bacteria, viruses, fungi, and venomous animals. Collectively, these diseases affect more than 1 billion people worldwide and remain prevalent in regions with limited access to sanitation, healthcare, and education, perpetuating social and economic disparities. Many NTDs are targeted for elimination as public health problems by 2030, in accordance with the United Nations Sustainable Development Goals (SDG 3.3) [1]. Achieving this goal requires not only improved prevention and surveillance but also the discovery of novel antiparasitic molecules capable of overcoming current therapeutic limitations [2, 3]. Despite their global relevance, few advances have been achieved in chemotherapy over the last four decades, and treatment still relies on a small number of drugs such as praziquantel, benznidazole, and amphotericin B [4]. Limitations in efficacy, adverse effects, and emerging resistance highlight the urgent need for new, safe, and affordable compounds capable of addressing both parasite viability and host‐driven pathological processes [5–7].

**Figure 1:**
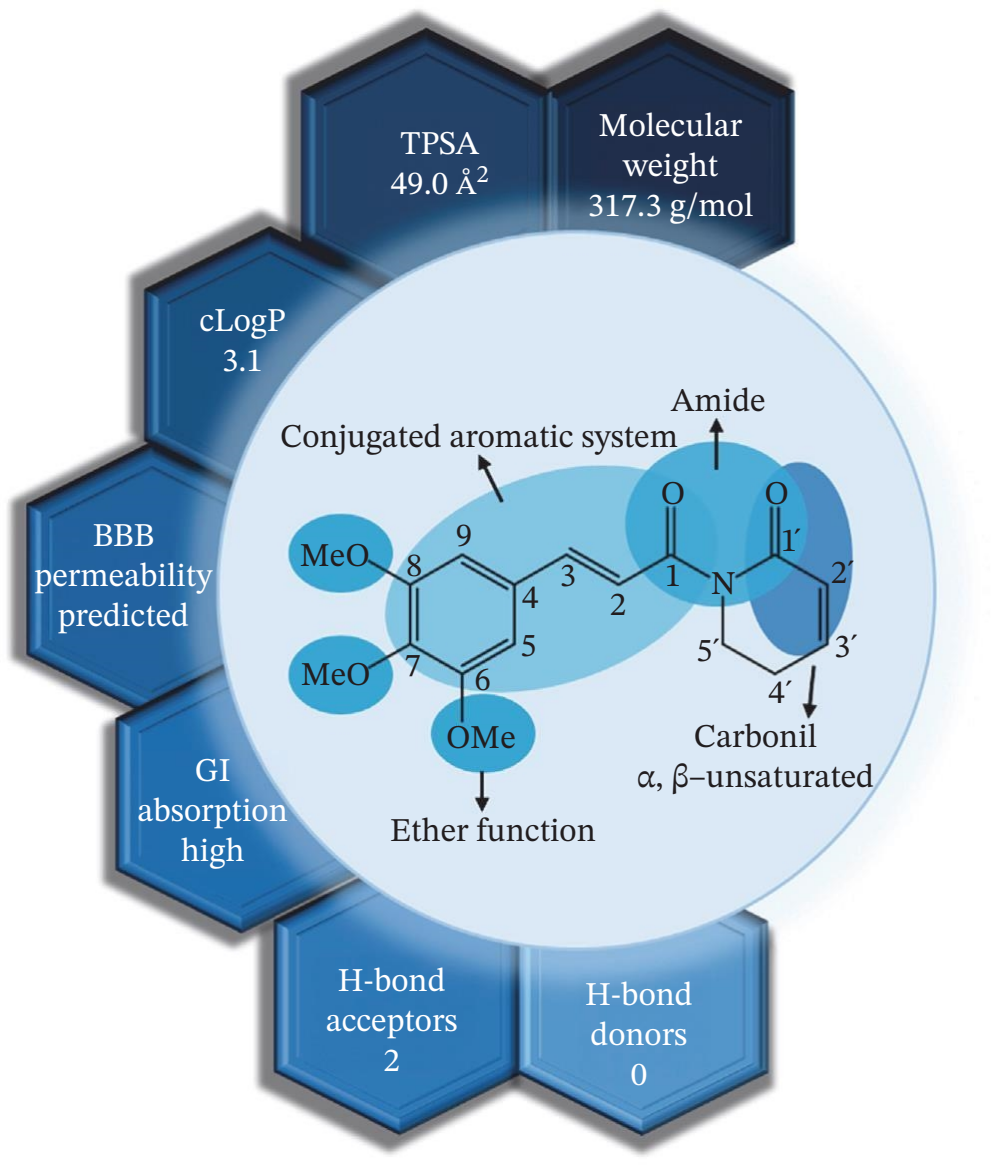
Chemical structure and physicochemical profile of piplartine (piperlongumine). The α,β‐unsaturated lactam and conjugated amide system confer electrophilic reactivity and redox sensitivity, underlying its biological activity. Physicochemical parameters were predicted using SwissADME and support its drug‐like properties and oral bioavailability.

Natural products remain a prolific source of bioactive molecules with potential applications in parasitic and immune‐mediated diseases [8]. Their structural diversity and evolutionary optimization often result in compounds capable of acting on multiple cellular targets. Among these, alkaloids and amides derived from *Piper* species (*Piperaceae*) have long been recognized for their antimicrobial, antiparasitic, and anti‐inflammatory properties [9]. Piplartine, also known as piperlongumine, is one such amide that has gained increasing attention for its broad pharmacological profile [10, 11]. It is isolated mainly from *P. longum*, *P. tuberculatum*, and *P. truncatum*, plants widely distributed in tropical regions [9]. Structurally, piplartine possesses a conjugated α,β‐unsaturated lactam system that confers electrophilic reactivity and redox sensitivity, both directly associated with its biological activity (Figure 1).

Among parasitic NTDs discussed in this review, schistosomiasis affects more than 250 million people worldwide, with over 700 million individuals at risk of infection, particularly in sub‐Saharan Africa, South America, and Southeast Asia [12]. Leishmaniasis remains endemic in nearly 100 countries, with an estimated 1 million new cases annually and a substantial burden of cutaneous and visceral disease [13]. Chagas disease affects ~6–7 million people globally, primarily in Latin America, and continues to expand through migration and congenital transmission [14]. Human African trypanosomiasis, although currently reported at historically low incidence, remains endemic in several countries of sub‐Saharan Africa, with an estimated 55 million people living in areas at risk of infection [15]. Although not formally classified as an NTD, malaria continues to represent a major global parasitic disease, with more than 240 million cases and over 600,000 deaths reported annually, predominantly in low‐ and middle‐income countries [16]. Together, these infections impose a disproportionate burden on vulnerable populations and underscore the urgent need for novel, broadly effective antiparasitic strategies.

Despite this substantial and well‐documented disease burden, advances in antiparasitic drug discovery have not progressed uniformly across parasite groups. Research efforts have historically concentrated on protozoan pathogens such as *Leishmania* spp. and *Trypanosoma* spp., driven in part by the availability of established screening platforms and validated molecular targets [2, 5]. In contrast, helminth infections, particularly those caused by trematodes and nematodes, have received comparatively less attention, resulting in a limited diversity of chemical scaffolds evaluated against these pathogens.

Within this context, *Schistosoma mansoni* remains the most extensively studied helminth species [17, 18], while *Angiostrongylus cantonensis*, the etiological agent of eosinophilic meningitis, has only recently emerged as a relevant experimental model for the discovery and validation of novel anthelmintic candidates [19–23]. This asymmetry highlights a persistent gap in helminth‐focused drug discovery and underscores the need for bioactive compounds capable of bridging protozoan‐ and helminth‐oriented research pipelines.

The antiparasitic potential of piplartine has been demonstrated in a range of species relevant to human disease. Studies revealed potent in vitro and in vivo effects against *S. mansoni* and *A. cantonensis*, reducing worm viability, oviposition, and tegument integrity [24–26]. The compound also exhibits strong activity against kinetoplastid protozoa, including *Leishmania* and *Trypanosoma*, where it disrupts parasite redox homeostasis, induces mitochondrial dysfunction, and promotes apoptosis‐like death. Collectively, these findings indicate that piplartine acts through mechanisms conserved across phylogenetically distinct parasites, supporting its classification as a broad‐spectrum antiparasitic scaffold [27, 28].

Beyond its direct parasiticidal action, piplartine exerts pronounced immunomodulatory and anti‐inflammatory effects, primarily described in mammalian immune and inflammatory models [29, 30]. It regulates oxidative and inflammatory signaling pathways that are central to host defense and tissue injury. In macrophages and dendritic cells, piplartine decreases the production of proinflammatory cytokines such as TNF‐α, IL‐1β, and IL‐6 while enhancing antioxidant responses through activation of the Nrf2–HO‐1 axis [31, 32]. It also inhibits NF‐κB activation and selectively targets the immunoproteasome, thereby modulating antigen processing and inflammatory gene expression. In T cells, piplartine induces redox‐dependent differentiation toward a regulatory phenotype, increasing IL‐10 production and FOXP3 expression. These properties suggest a compound capable of rebalancing immune responses and potentially mitigating immunopathology, although their direct contribution during parasitic infection remains to be fully elucidated.

This review summarizes current knowledge on piplartine as a natural product with dual antiparasitic and immunomodulatory properties. We compile experimental evidence from studies on *S. mansoni*, *Leishmania* spp., *T. cruzi*, *T. brucei*, and other parasites of medical importance, highlighting how its redox‐regulated mechanisms contribute to parasite killing and immune regulation. The discussion extends to host inflammatory pathways, focusing on NF‐κB inhibition, Nrf2 activation, and selective immunoproteasome modulation as central elements of piplartine’s pharmacological profile. Finally, we explore translational perspectives for developing piplartine and its derivatives as multitarget scaffolds for antiparasitic and immune‐directed therapeutic strategies.

## 2. Antiparasitic Activity of Piplartine Against Helminths and Protozoa

The discovery of natural products with dual antiparasitic and immunomodulatory potential has attracted significant attention in the search for new therapeutics against NTDs [33–36]. Piplartine exhibits a broad spectrum of antiparasitic activity across phylogenetically diverse helminths and protozoa while maintaining a favorable safety profile in mammalian cells. Its chemical simplicity, redox‐reactive pharmacophore, and biological versatility support its classification as a lead‐like natural scaffold for further pharmacological and medicinal chemistry development.

**Figure 2:**
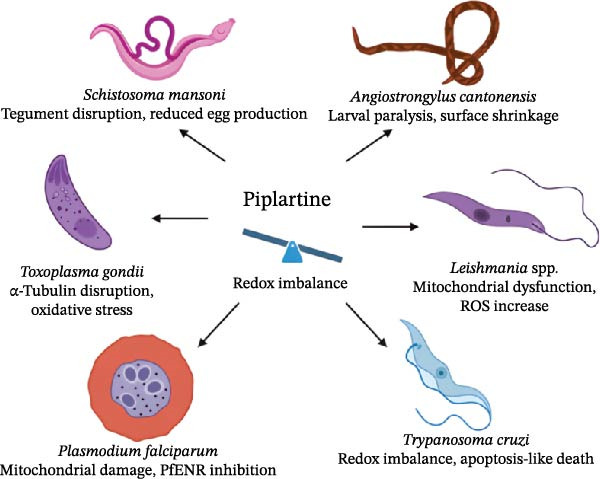
Antiparasitic spectrum and mechanistic targets of piplartine. Schematic overview showing piplartine’s activity against helminths (*S. mansoni* and *A. cantonensis*) and protozoa (*Leishmania*, *T. cruzi*, *T. brucei*, *P. falciparum*, and *T. gondii*). Arrows indicate the main biological effects observed for each parasite group, including tegumental disruption, mitochondrial dysfunction, and oxidative stress–related damage. Central nodes emphasize the common mechanism of redox imbalance driving parasite death.

Comprehensive studies have evaluated the biological activity of piplartine through in vitro and in vivo assays involving diverse parasitic models. In vitro evaluations have established the compound’s efficacy against both helminths and protozoa, with low‐micromolar EC_50_ or IC_50_ values and selective toxicity toward parasites over mammalian cells (Table 1). In vivo investigations in murine models have further confirmed its antiparasitic potential, demonstrating significant reductions in worm burden or lesion size following oral or intralesional administration (Table 2). Together, these findings underscore the translational relevance of piplartine and its derivatives as promising prototypes for the treatment of neglected and emerging parasitic diseases (Figure 2).

**Table 1:** In vitro antiparasitic activity and selectivity of piplartine and its analogs against helminths and protozoa.

Parasite species	Compound	Parasite stage	EC_5_₀/IC_5_₀ (µM)	Cell line (cytotoxicity)	CC_5_₀ (µM)	Selectivity index (SI)	References
*S. mansoni*	Piplartine	Adult worms	6.3	Vero	>31.5	–	[24]
*S. mansoni*	Piplartine	Schistosomula	15	–	–	–	[37]
*A. cantonensis*	Piplartine	L1/L3 larvae	8.3/10.4	–	>500	–	[26]
*L. amazonensis* (IFLA/BR/1967/PH8 strain)	Piplartine	Promastigote/amastigote	3.3/0.4	J774	10	~25	[38]
*L. amazonensis* (MHOM/BR/pH8 strain)	Piplartine	Promastigote	564.1	Peritoneal cells	731.1	1.3	[39]
*L. braziliensis*	Piplartine	Promastigote/amastigote	8.5/1.4	HepG2, J774, THP‐1, and Vero	29.5/8.8	20/6	[40]
*L. guyanensis*	Piplartine	Promastigote	2.2	J774	10.4	4.7	[28]
*L. infantum*	Piplartine	Promastigote/amastigote	7.9/8.2	Macrophages	10	25	[38]
*L. donovani*	Piplartine	Promastigote	7.5	–	–	–	[41]
*L. major*	Piplartine analogs	Promastigote	4.5–5.8	Macrophages	115	~20	[42]
*P. falciparum*	Piplartine	Intraerythrocytic stages	10.1–19.5	Peritoneal cells	731.1	37–72	[39]
*T. brucei*	Piplartine analogs	Amastigote/epimastigote	10–12	BS442 cells	–	–	[42]
*T. cruzi*	Piplartine	Epimastigote/amastigote	53.8/33.9	LLCMK₂	303	8.9	[43]
*T. cruzi* (*Y* strain)	Compound 17 (benzamide derivative)	Trypomastigote/epimastigote/amastigote	2.2/8.7/1.7	Vero	657	~300	[44]
*T. gondii*	Piplartine analogs	Tachyzoite	2.0–2.2	Vero	23.6–26.4	11–13	[42]

*Note:* Summary of in vitro antiparasitic activity of piplartine and selected analogs against helminths and protozoa. The table reports parasite species, tested stage, effective concentration (EC_5_₀/IC_5_₀), cytotoxic concentration (CC_5_₀), and selectivity index (SI). Values indicate broad‐spectrum efficacy and preferential toxicity toward parasites over mammalian cells.

**Table 2:** In vivo antiparasitic efficacy of piplartine in murine models of helminth and protozoan infections.

Animal model	Parasite species	Dose (mg/kg)	Route of administration	Antiparasitic activity	References
Mouse (*Mus musculus*)	*Schistosoma mansoni*	400	Oral (p.o.)	60.4% reduction in worm burden; decreased egg production	[25]
Mouse (*Mus musculus*)	*Leishmania amozonensis*	25 (4 days)	Intralesional (i.l.)	40% reduction in lesion size; 55% decrease in parasite load	[28]
Hamster (*Mesocricetus auratus*)	*Leishmania donovani*	30 (10 days, 5‐day interval)	Intraperitoneal (i.p.)	36% reduction in parasite burden; 50% reduction	[41]

*Note:* This table summarizes the in vivo antiparasitic activity of piplartine evaluated in animal models of helminth and protozoan infections. Reported outcomes include reductions in parasite burden, egg production, tissue pathology, and other disease‐related parameters, depending on the experimental model and infection stage assessed. Efficacy should be interpreted in the context of dosing regimen, route of administration, treatment duration, and host species. Collectively, the data illustrate the in vivo efficacy of piplartine across distinct parasitic infections while highlighting differences in experimental design that may influence direct quantitative comparisons between studies.

### 2.1. Schistosoma mansoni

*Schistosoma mansoni*, the causative agent of schistosomiasis, has been the most extensively investigated helminth in studies of piplartine, both in vitro and in vivo (Tables 1 and 2). The compound exhibits potent activity against multiple developmental stages of *S. mansoni* with remarkable selectivity toward the parasite.

In vitro, piplartine caused complete mortality of adult worms at 15.8 µM within 24 h and produced a 75% reduction in oviposition at 6.3 µM. Microscopic analysis revealed severe tegumental damage, including tubercle collapse, spine disorganization, and sucker deformation, whereas Vero mammalian cells remained unaffected, indicating a high selectivity index (SI) (>13) [24]. Piplartine also displayed strong activity against schistosomula at concentrations between 7.5 and 15 µM, inducing granular tegumental surfaces and reduced body length in a dose‐ and time‐dependent manner [37]. These morphological alterations were not observed in parasites treated with praziquantel under the same experimental conditions, suggesting distinct or complementary mechanisms of action.

Synergistic assays further demonstrated the potential of piplartine in combination therapies. When coadministered with the antimicrobial peptide dermaseptin, complete mortality of both adult and immature *S. mansoni* occurred within 72 h, accompanied by irreversible inhibition of oviposition. Confocal microscopy confirmed extensive tegumental erosion and increased surface permeability, consistent with synergistic membrane disruption [45]. These results emphasize the potential of piplartine‐based combinations to enhance therapeutic efficacy and reduce the likelihood of resistance development.

Additional in vitro combination studies demonstrated that piplartine also acts synergistically with praziquantel and the alkaloid epiisopiloturine against adult *S. mansoni* [46]. Fixed‐ratio assays revealed strong synergy for piplartine + praziquantel, with 100% mortality at concentrations where each compound alone was inactive (1.98 µM piplartine + 0.63 µM praziquantel; combination index ≈ 0.42), and pronounced tegumental disruption under confocal and scanning electron microscopy. Piplartine + epiisopiloturine produced similar synergistic profiles (combination index ≈ 0.61) with extensive surface erosion and reduced cytotoxicity toward mammalian cells (Vero, L929, and MDCK). These results confirm the potential of piplartine as a synergistic partner capable of enhancing efficacy and reducing host toxicity in antischistosomal combinations.

Complementary structure–activity analyses compared piplartine with five synthetic analogs against adult *S. mansoni* and mammalian cell lines [47]. Under the same conditions, piplartine killed adult worms at 5–10 µM, whereas most analogs were inactive. Several derivatives exhibited reduced cytotoxicity in NIH‐3T3 fibroblasts and J774A.1 macrophages, with apoptosis as the predominant cell‐death phenotype. Structure–activity relationship data highlighted the essential role of the α,β‐unsaturated chain and the dihydropiperidinone and trimethoxybenzene moieties for schistosomicidal activity; modifications in these features diminished efficacy while often lowering host‐cell toxicity. These findings identify structural elements critical for activity and inform future design of safer piplartine‐based analogs.

The in vivo efficacy of piplartine was confirmed using a murine model of schistosomiasis. Oral administration at 400 mg/kg resulted in a 60% reduction in worm burden and a 65% reduction in egg production, while treatment during the prepatent phase achieved a 54% decrease in worm recovery. Scanning electron microscopy revealed marked tegumental damage characterized by spine loss and blister formation. The reduction in egg output suggests that piplartine may also attenuate granulomatous inflammation and other pathological outcomes associated with schistosomiasis (Table 2).

Collectively, these findings demonstrate that piplartine exerts potent schistosomicidal effects across multiple parasite stages, combining tegumental disruption, interference with neuromuscular function, and possible modulation of host pathology. Its high selectivity and oral efficacy underscore its potential as a prototype for next‐generation antischistosomal agents and as a candidate for combination regimens with existing drugs.

### 2.2. Angiostrongylus cantonensis

The anthelmintic potential of piplartine extends beyond trematodes, encompassing the neurotropic nematode *A. cantonensis*, the causative agent of eosinophilic meningitis [26]. The compound displayed potent larvicidal activity against both first‐stage (L1) and infective third‐stage (L3) larvae, with EC_50_ values of 8.3 and 10.4 µM, respectively, outperforming albendazole under comparable conditions. Morphological analyses revealed marked tegumental disruption, caudal shrinkage, and loss of motility, consistent with severe damage to the larval surface.

Piplartine exhibited remarkable selectivity toward the parasite, as it showed no detectable toxicity to the nonparasitic nematode *Caenorhabditis elegans* at concentrations up to 500 µM, whereas albendazole induced high mortality at 14.8 µM [26]. This differential response highlights the safety margin of piplartine and suggests a distinct mechanism of action from benzimidazole anthelmintics.

### 2.3. Leishmania spp.

*Leishmania* species, the etiological agents of leishmaniasis, comprise more than 20 protozoan parasites that cause cutaneous, mucocutaneous, and visceral forms of the disease. The leishmanicidal activity of piplartine has been extensively reported across multiple *Leishmania* species, including *L. amazonensis*, *L. braziliensis*, *L. donovani*, *L. guyanensis*, and *L. infantum*. The compound exhibits broad‐spectrum efficacy in vitro and in vivo, as summarized in Tables 1 and 2.

In early studies, piplartine inhibited *L. donovani* promastigotes with an IC_50_ of 7.5 µM and significantly reduced spleen parasite burden in infected hamsters after intraperitoneal administration of 30 mg/kg for 10 days, resulting in a 36% reduction in parasite load and 50% decrease in spleen weight. No hepatic toxicity was observed, confirming its tolerability in vivo [41].

Subsequent investigations expanded these findings to other *Leishmania* species. In *L. amazonensis* (IFLA/BR/1967/PH8 strain) and *L. infantum*, piplartine exhibited potent in vitro activity, with IC_50_ values of 3.3 and 7.9 µM for promastigotes and 0.4 µM for intracellular amastigotes. The compound showed a CC_50_ of 10 µM in J774 macrophages, corresponding to a SI of ~25 [38]. Maintenance of the α,β‐unsaturated carbonyl and lactam moieties was essential for activity, while microsomal stability and predicted oral bioavailability supported its drug‐like potential.

In a broader screening using piplartine isolated from *Piper pseudoarboreum*, the compound exhibited strong and comparable potency across *L. amazonensis*, *L. braziliensis*, *L. guyanensis*, and *L. infantum*, with IC_50_ values ranging from 1.6 to 3.8 µM for promastigotes and 8.2 µM for intracellular amastigotes [48]. In a murine model of cutaneous leishmaniasis, intralesional administration of 25 mg/kg for four consecutive days reduced lesion size by 40% and parasite load by 55%, achieving efficacy comparable to glucantime [28].

Mechanistic studies with *L. braziliensis* revealed that piplartine increases reactive oxygen species (ROS) generation, disrupts membrane integrity, and induces lipid body accumulation. The compound inhibited trypanothione reductase, a key redox enzyme, confirming that oxidative imbalance contributes to parasite death [40]. Similar findings were reported for *L. guyanensis* and *L. infantum*, where oxidative stress–related pathways were implicated in parasite killing [28, 48]. These results are consistent with the electrophilic α,β‐unsaturated lactam structure of piplartine and its ability to disturb parasite redox homeostasis.

Earlier comparative analyses among Piper‐derived amides reported more modest potency for *L. amazonensis* (MHOM/BR/pH8 strain; IC_50_ = 564.1 µM), but structural modifications, particularly aromatic substitutions at the amide moiety, markedly improved activity, highlighting the importance of hydrophobic substituents for target engagement [39]. Expanded screening of Piper‐based amides also confirmed low‐micromolar potency against *L. major* promastigotes (IC_50_ = 4.5–5.8 µM), with a marked loss of activity upon disruption of the conjugated enone/lactam motif [42].

Recent medicinal chemistry efforts have sought to optimize piplartine derivatives to improve potency and selectivity. Nóbrega and colleagues [49] synthesized a series of piplartine‐based esters and amides bearing aromatic or methoxy substituents. Several derivatives exhibited remarkable activity against *L. amazonensis* promastigotes, with IC_50_ values as low as 0.025 µM and favorable selectivity toward mammalian cells. Structure–activity relationship analyses indicated that increased lipophilicity and electron‐donating groups at the aromatic ring enhanced leishmanicidal potency, likely by facilitating membrane permeation and intracellular accumulation. Molecular docking analyses suggested interactions with trypanothione reductase and other redox enzymes, reinforcing oxidative stress as a unifying mechanism of action.

### 2.4. Trypanosoma spp.

*Trypanosoma cruzi*, the etiological agent of Chagas disease (American trypanosomiasis), and *Trypanosoma brucei*, the causative agent of African trypanosomiasis (sleeping sickness), are kinetoplastid protozoa that cause major parasitic infections in Latin America and Africa, respectively. The trypanocidal activity of piplartine has been extensively documented through morphological, biochemical, and mechanistic analyses (Table 1). The compound inhibits *T. cruzi* proliferation and induces profound ultrastructural changes, including mitochondrial swelling, ‐vacuolization, and nuclear disorganization, leading to loss of membrane integrity and apoptosis‐like death. Cytotoxicity assays confirmed low toxicity toward mammalian cells, demonstrating a clear selectivity window compared with benznidazole [43].

Proteomic and biochemical investigations performed in two distinct *T. cruzi* strains demonstrated that piplartine treatment alters the expression of redox‐regulatory enzymes such as tryparedoxin peroxidase and methionine sulfoxide reductase, resulting in the accumulation of ROS and mitochondrial dysfunction. These findings indicate that the compound interferes with thiol‐dependent antioxidant systems that are essential for parasite survival [27].

Complementary evaluations of other Piper‐derived amides confirmed consistent trypanocidal activity with minimal cytotoxicity in mammalian cells [50]. Across independent studies, piplartine exhibited IC_50_ values ranging from 4 to 8 µM against epimastigote and intracellular amastigote forms of *T. cruzi*, with CC_50_ values above 200 µM in Vero cells, corresponding to selectivity indices exceeding 25 [27, 43]. Mechanistic analyses revealed mitochondrial depolarization, redox imbalance, and inhibition of ATP generation, reinforcing oxidative stress as a central determinant of trypanocidal activity [27, 42, 43].

Recent studies have consolidated these observations, linking the antitrypanosomal effect of piplartine to inhibition of antioxidant enzymes, collapse of mitochondrial potential, and redox imbalance [51]. Together, these effects contribute to parasite death and underscore the compound’s potential as a prototype for new agents against Chagas disease.

Beyond *T. cruzi*, piplartine and its analogs also display activity against *T. brucei*. Screening assays revealed IC_50_ values around 10–12 µM for amastigote and epimastigote forms, confirming sensitivity comparable to that observed for *T. cruzi* [42]. Although detailed mechanistic data for *T. brucei* remain limited, the observed redox reactivity of the compound suggests a conserved mode of action involving mitochondrial dysfunction and oxidative stress across kinetoplastids.

Medicinal chemistry advances have further optimized the piplartine scaffold. Silva and collaborators [52] synthesized 23 benzamide and ester derivatives and evaluated them against trypomastigote, epimastigote, and amastigote forms of *T. cruzi*. Among them, *N*‐isobutyl‐3,4,5‐trimethoxybenzamide (compound **17**) displayed the highest activity, with an IC_50_ of 2.2 µM against trypomastigotes, a CC_50_ of 657 µM in Vero cells, and an exceptional SI of ~300. Microscopic analyses revealed mitochondrial depolarization and morphological damage consistent with mixed necrotic and apoptotic mechanisms. In silico modeling identified histone deacetylase (HDAC) as a potential molecular target, suggesting that the electrophilic core of piplartine may influence both redox and epigenetic regulation in *T. cruzi*. These findings demonstrate that rational modifications of the piplartine nucleus can yield highly potent trypanocidal agents while maintaining favorable selectivity and mechanistic profiles.

### 2.5. Other Protozoan Parasites

Although most studies on piplartine have focused on parasites officially classified as NTDs, additional evidence indicates that this compound also exhibits potent activity against other protozoa of major medical relevance, notably *Plasmodium falciparum* and *Toxoplasma gondii* (Table 1). *P. falciparum*, the causative agent of malaria, and *T. gondii*, responsible for toxoplasmosis, share conserved redox and metabolic pathways with kinetoplastid and helminth parasites, making them valuable models for investigating the general antiparasitic mechanisms of piplartine and its synthetic derivatives.

In *P. falciparum*, piplartine inhibited intraerythrocytic growth with IC_50_ values in the low‐micromolar range (10.1–19.5 µM), producing morphological changes such as vacuolization, pigment loss, and mitochondrial swelling, hallmarks of redox stress and energy depletion [39]. The compound exhibited low cytotoxicity toward murine peritoneal cells (CC_50_ = 731 µM), resulting in selectivity indices between 37 and 72, consistent with a favorable safety margin. Computational and biochemical studies further demonstrated that piplartine and several of its analogs interact with *P. falciparum* enoyl‐acyl carrier protein reductase (PfENR), an essential enzyme in the fatty acid synthesis pathway, supporting the role of redox imbalance in parasite death [44]. Derivatives bearing halogen or methoxy substituents displayed enhanced potency and selectivity, reinforcing the structural importance of the α,β‐unsaturated amide system for biological activity [39, 44].

Comparable effects have been reported in *T. gondii*, where piplartine and newly synthesized analogs significantly reduced tachyzoite proliferation in vitro. Halogenated derivatives exhibited higher efficacy and selectivity than the parent compound, with IC_50_ values between 3 and 10 µM. Microscopy revealed mitochondrial swelling and cytoplasmic disorganization consistent with apoptosis‐like processes, while molecular docking identified α‐tubulin and redox‐regulatory enzymes as potential targets. These results suggest that piplartine disrupts cytoskeletal organization and redox homeostasis in *T. gondii*, mirroring the mechanisms observed in kinetoplastid parasites. The susceptibility of *T. gondii* to piplartine thus broadens its recognized antiparasitic spectrum and supports its potential as a versatile chemical scaffold for developing pan‐protozoan therapeutics [42].

## 3. Pharmacokinetic and Drug‐Likeness Properties

In addition to its antiparasitic and immunomodulatory activities, piplartine displays physicochemical and pharmacokinetic attributes consistent with early‐stage drug‐likeness [26]. While the chemical structure and selected physicochemical features of piplartine are illustrated in Figure 1, the parameters discussed here are specifically interpreted in the context of pharmacokinetic behavior and drug‐likeness. According to in silico analyses performed using SwissADME and related predictive platforms, piplartine has a molecular weight of ~317.3 g/mol, a calculated LogP (cLogP) of ~ 2.4–2.6, and a topological polar surface area (TPSA) of ~47 Å^2^ (Table 3). The molecule contains one hydrogen bond donor (HBD) and four hydrogen bond acceptors (HBAs), fully complying with Lipinski’s rule of five as well as Veber, Egan, and Ghose criteria.

**Table 3:** Pharmacokinetic and drug‐likeness features of piplartine.

Parameter	Predicted value	Interpretation for drug‐likeness	References
Molecular weight (g/mol)	317.3	Within the optimal range (<500 g/mol) for oral drug candidates	[10, 26]
cLogP	2.4–2.6	Moderate lipophilicity, compatible with membrane permeability and oral absorption	[26]
Topological polar surface area (TPSA, Å²)	~47	Below the 140 Å² threshold, supporting good intestinal absorption	[26]
Hydrogen bond donors (HBD)	1	Compliant with Lipinski’s rule of five	[26]
Hydrogen bond acceptors (HBAs)	4	Compliant with Lipinski’s rule of five	[26]
Lipinski’s rule of five	No violations	Indicates favorable oral drug‐likeness	[26]
Veber rule	Compliant	Suggests adequate molecular flexibility and oral bioavailability	[26]
Egan rule	Compliant	Supports passive membrane permeability	[26]
Ghose filter	Compliant	Indicates physicochemical suitability for drug development	[26]
Gastrointestinal absorption (in silico)	High (~94%)	Predicts favorable oral exposure	[26]
P‐glycoprotein (P‐gp) substrate	No	Suggests low efflux liability	[26]
Blood–brain barrier permeability	Predicted	Potential relevance for neurotropic infections	[26]
Acute toxicity (LD_5_₀, rodents)	>3000 mg/kg	Indicates low acute systemic toxicity	[10]

*Note:* Predicted physicochemical and pharmacokinetic parameters of piplartine derived primarily from in silico analyses (SwissADME) and experimental toxicity studies. Compliance with Lipinski, Veber, Egan, and Ghose criteria supports the classification of piplartine as a lead‐like natural scaffold with properties compatible with oral exposure and further preclinical optimization. Experimental validation of absorption, distribution, metabolism, excretion, and long‐term immunological safety remains necessary for translational advancement.

Predicted gastrointestinal (GI) absorption is high (≈94%), and piplartine is not classified as a P‐glycoprotein (P‐gp) substrate, suggesting a favorable profile for oral exposure with limited efflux liability. In silico models also indicate potential blood–brain barrier (BBB) permeability, which may be relevant for neurotropic parasitic infections such as angiostrongyliasis. Importantly, these pharmacokinetic parameters are derived primarily from computational predictions and should be interpreted as indicative rather than confirmatory.

Experimental pharmacokinetic data for piplartine remain limited. To date, no comprehensive in vivo absorption, distribution, metabolism, and excretion (ADME) studies have been reported in parasitic infection models. Nevertheless, available toxicological data indicate low acute systemic toxicity, with reported LD_50_ values exceeding 3000 mg/kg in rodent models following oral administration [10]. While these findings support an acceptable preliminary safety margin, they do not exclude the possibility of cumulative toxicity or immunosuppressive effects following repeated or long‐term exposure.

Collectively, the predicted drug‐likeness properties of piplartine support its classification as a lead‐like natural product suitable for further optimization. However, advancing this scaffold toward translational applications will require rigorous experimental validation of oral bioavailability, metabolic stability, tissue distribution, and immunological safety, particularly in the context of chronic parasitic infections.

## 4. Immunomodulatory and Anti‐Inflammatory Properties of Piplartine

**Figure 3:**
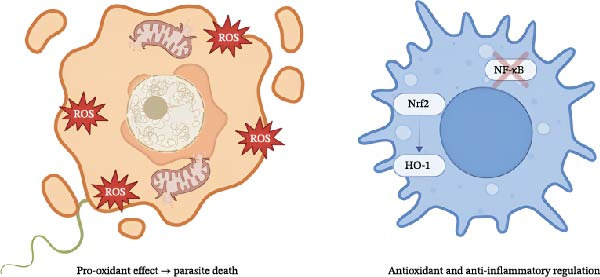
Dual redox modulation of piplartine in parasite and host immune cells. The left panel (red tones) represents the prooxidant activity of piplartine in parasites, where it induces excessive reactive oxygen species (ROS), mitochondrial damage, and apoptosis. The right panel (blue tones) illustrates its antioxidant and anti‐inflammatory actions in host immune cells, where it activates the Nrf2–HO‐1 pathway and suppresses NF‐κB–mediated inflammation, restoring redox balance and immune homeostasis.

Piplartine exerts complex regulatory effects on oxidative and inflammatory signaling pathways that are central to immune activation and tissue injury. Its dual behavior, prooxidant in target cells and antioxidant in host tissues, underlies its therapeutic versatility (Figure 3). By modulating redox‐sensitive transcription factors such as NF‐κB and Nrf2, the compound influences cytokine production, macrophage activation, and T cell differentiation. In macrophages, piplartine suppresses TNF‐α–induced NF‐κB activation by preventing nuclear translocation of the p65 subunit and directly interacting with key upstream regulators including IκBα, IKKα, p50, p65, and TAK1 [29]. These interactions block IκBα phosphorylation and degradation, attenuating downstream cytokine release and oxidative damage (Table 4).

**Table 4:** Immunomodulatory mechanisms of piplartine in mammalian systems.

Model/cell type	Main effects	Molecular pathway	References
Macrophages	↓ TNF‐α, IL‐6, IL‐1β; ↑ IL‐10	Inhibition of TNF‐α–induced NF‐κB activation via interaction with IκBα, IKKα, p50, p65, and TAK1; Nrf2 activation and HO‐1 induction	[29]
Primary T cells	↓ IL‐17, IFN‐γ; ↑ FOXP3	ROS accumulation and GSH depletion leading to Th17→Treg shift	[53]
Dendritic cells	↓ CD80, CD86; ↑ GSTP1, CBR1	Inhibition of oxidative maturation signaling; suppression of antigen presentation	[30]
Activated macrophages/epithelial cells	↓ COX‐2, iNOS, TNF‐α, ICAM‐1	Selective inhibition of immunoproteasome β5i; NF‐κB suppression via IKK degradation	[54, 55]
Epithelial and carcinoma models	↓ IL‐6, ZEB1, Slug; ↑ E‐cadherin, miR‐200c	Inhibition of Akt/mTOR and EMT‐related transcription factors; activation of caspase‐9 and PARP	[56, 57]
Multiple immune cell types	↓ p38, JNK, p65 phosphorylation; ↑ HO‐1, SOD	Inhibition of MAPK and PI3K/Akt pathways; activation of Nrf2‐dependent antioxidant response	[29, 58]

*Note:* Summary of immunomodulatory and anti‐inflammatory effects of piplartine and selected analogs. The table includes key molecular targets, signaling pathways, and regulatory outcomes across distinct immune and epithelial models, illustrating the compound’s multitarget redox‐dependent mechanism of action.

### 4.1. Redox‐Dependent Modulation of Immune Cells

In immune cells, piplartine functions as a redox‐dependent immunomodulator, as demonstrated across multiple independent studies in mammalian macrophages, dendritic cells, and T lymphocytes, predominantly evaluated in noninfectious inflammatory or autoimmune experimental models [29, 42, 53].

In primary human and murine T cells, piplartine suppresses activation and proliferation by inducing controlled ROS accumulation and transient glutathione depletion. This redox shift favors a phenotypic transition from proinflammatory Th17 cells toward a regulatory T cell (Treg) profile, characterized by reduced IL‐17 and IFN‐γ secretion and increased FOXP3 expression and IL‐10 production [53].

In macrophage models, piplartine downregulates the production of proinflammatory cytokines such as TNF‐α, IL‐6, and IL‐1β through inhibition of NF‐κB activation and concomitant activation of the Nrf2–HO‐1 antioxidant axis, contributing to attenuation of inflammatory signaling and restoration of redox balance [29].

Importantly, although these immunomodulatory effects may be relevant to parasitic diseases characterized by immune‐driven pathology, they have been described predominantly in noninfectious models, and their direct impact on host–parasite interactions during infection remains to be determined.

### 4.2. Selective Immunoproteasome Inhibition

Beyond redox‐sensitive signaling pathways, piplartine has been identified as a selective inhibitor of the immunoproteasome, adding an additional dimension to its immunomodulatory profile. Biochemical and cellular studies have demonstrated that piplartine preferentially targets the β5i (LMP7) catalytic subunit of the immunoproteasome while sparing the constitutive proteasome, thereby modulating inflammatory signaling without broadly impairing basal protein turnover [54].

Selective immunoproteasome inhibition by piplartine has been associated with reduced activation of NF‐κB–dependent transcriptional programs and decreased expression of proinflammatory mediators, including COX‐2, iNOS, TNF‐α, and adhesion molecules such as ICAM‐1 [54]. Structural derivatives, including the dihydroxy analog PL‐18, further enhance these effects by promoting IKK degradation and preventing nuclear translocation of the NF‐κB p65 subunit, resulting in attenuated secretion of cytokines and chemokines involved in leukocyte recruitment [55].

Importantly, immunoproteasome inhibition represents a context‐dependent immunological mechanism. While attenuation of excessive inflammatory signaling may be beneficial in chronic inflammatory or immune‐mediated conditions, excessive suppression of immunoproteasome activity could theoretically impair antigen processing and presentation, potentially compromising adaptive immune responses required for effective parasite control. To date, the immunological consequences of piplartine‐mediated immunoproteasome inhibition have not been evaluated in parasite‐infected models, and its impact on host–parasite interactions remains to be determined.

### 4.3. Effects on Dendritic Cell Function and Antigen Presentation

Evidence from experimental studies indicates that piplartine modulates dendritic cell function primarily through redox‐dependent mechanisms, leading to attenuation of inflammatory activation rather than direct cytotoxicity [30].

In murine and human dendritic cell models, piplartine suppresses dendritic cell maturation by reducing intracellular ROS levels and limiting oxidative signaling pathways required for full activation. This effect is associated with downregulation of costimulatory molecules such as CD80 and CD86 and reduced production of proinflammatory cytokines, resulting in diminished antigen‐presenting capacity and impaired stimulation of antigen‐specific T cell responses [30].

In vivo evidence supporting these effects derives from noninfectious inflammatory models. In murine collagen‐induced arthritis, piplartine treatment reduced splenic dendritic cell maturation, collagen‐specific CD4^+^ T cell responses, and systemic inflammatory cytokine levels, leading to attenuation of joint inflammation and tissue damage [30].

Importantly, although modulation of dendritic cell activation may be beneficial in pathological inflammatory conditions, these effects have been described predominantly in noninfectious settings. The impact of piplartine‐mediated dendritic cell regulation on antigen presentation, protective immunity, and parasite clearance during parasitic infection remains to be determined.

### 4.4. Broader Regulation of Inflammatory Signaling

Complementary evidence from epithelial and cancer models reinforces the broad anti‐inflammatory spectrum of piplartine. The compound suppresses IL‐6 synthesis and epithelial–mesenchymal transition by rebalancing redox homeostasis and inhibiting transcription factors ZEB1 and Slug while restoring E‐cadherin and miR‐200 c expression [56]. In thyroid carcinoma cells, it downregulates IL‐6 and antiapoptotic proteins through inhibition of Akt/mTOR and activation of caspase‐9 and PARP, confirming its redox‐dependent control over inflammation and cell survival [57]. Additionally, microtubule destabilization induced by piplartine interferes with mitotic and stress‐response signaling, reinforcing its ROS‐linked regulation of inflammatory cascades [59].

### 4.5. MAPK, PI3K/Akt, and Nrf2 Signaling Integration

Piplartine also modulates upstream kinases involved in inflammatory transduction. It inhibits phosphorylation of p38, JNK, and NF‐κB p65 while sparing ERK activity, thereby selectively reducing transcription of proinflammatory cytokines and chemokines and limiting leukocyte recruitment [54, 58]. Simultaneously, Nrf2 activation upregulates antioxidant defenses, including HO‐1 and SOD, protecting tissues from oxidative injury and inflammation [29].

### 4.6. Integrative Immunopharmacological Model

Collectively, these findings define piplartine as a multifunctional redox‐regulated immunopharmacological agent [30, 54, 55]. It induces oxidative stress in parasites and tumor cells while restoring redox balance and dampening inflammation in host tissues. The convergence of immunoproteasome inhibition, NF‐κB suppression, Nrf2 activation, and dendritic cell reprograming positions piplartine as a promising natural scaffold for developing multitarget therapies that address both infection‐driven and immune‐mediated inflammatory diseases.

### 4.7. Linking Immunomodulation to Parasite Control: Opportunities and Limitations

The dual antiparasitic and immunomodulatory properties of piplartine raise important questions regarding how host‐directed immune regulation may intersect with parasite control and disease outcome. In parasitic infections, immune responses play a dual role, as appropriately coordinated inflammation contributes to parasite clearance, whereas excessive or dysregulated immune activation is a major driver of tissue damage and pathology. Consequently, immunomodulation may represent a therapeutic opportunity, but its impact is inherently context dependent.

Current evidence indicates that the primary antiparasitic activity of piplartine is mediated by direct effects on parasites, particularly through disruption of redox homeostasis, mitochondrial dysfunction, and metabolic imbalance. In contrast, immunomodulatory effects such as suppression of NF‐κB signaling, activation of the Nrf2–HO‐1 axis, selective immunoproteasome inhibition, and modulation of antigen‐presenting cell function have been characterized predominantly in noninfectious inflammatory or autoimmune models. As a result, direct cause–effect relationships between piplartine‐induced immune modulation and enhanced parasite clearance during infection have not yet been experimentally established.

From a translational perspective, host‐directed immunomodulation by piplartine may be beneficial in parasitic diseases in which immunopathology contributes substantially to morbidity, including schistosomiasis and chronic protozoan infections. However, excessive suppression of inflammatory or antigen‐presenting pathways could theoretically compromise protective immune mechanisms required for effective parasite elimination. Therefore, the net therapeutic impact of piplartine is likely to reflect a balance between direct parasiticidal activity and context‐dependent modulation of host immune responses.

Future studies integrating parasitological, immunological, and pharmacodynamic endpoints in infected animal models will be essential to clarify whether immunomodulation by piplartine primarily mitigates disease pathology, contributes to parasite control, or exerts both effects depending on infection stage and host immune status. Such investigations will be critical for defining the therapeutic window and translational relevance of piplartine and its derivatives as dual‐acting antiparasitic agents.

## 5. Translational Perspectives and Concluding Remarks

The pharmacological profile of piplartine reveals a convergence of antiparasitic, redox‐modulating, and immunoregulatory properties that support its classification as a lead‐like natural scaffold with translational potential, rather than a defined therapeutic agent, for NTDs and immune‐mediated disorders. Across distinct parasite groups, piplartine consistently exhibits potent activity against trematodes, nematodes, and kinetoplastid protozoa at low‐micromolar or submicromolar concentrations. Its broad efficacy against *S. mansoni*, *A. cantonensis*, *Leishmania* spp., and *T. cruzi* demonstrates that its action is not restricted to a single parasite lineage but rather reflects conserved mechanisms involving redox disruption and metabolic imbalance. The reduction of egg production and parasite burden in animal models of schistosomiasis and leishmaniasis indicates that piplartine affects both survival and reproduction, two critical parameters for transmission control and pathology reduction. However, the limited number of in vivo studies currently available underscores the need for expanded validation across different parasite taxa, infection stages, and experimental models.

The development of piplartine analogs through medicinal chemistry provides a rational path for optimizing potency and pharmacokinetic properties while retaining the redox‐reactive pharmacophore responsible for antiparasitic activity and immunomodulatory effects. Structural refinements, such as aromatic methoxylation and amide substitutions, have yielded derivatives with superior activity and selectivity against *Leishmania* spp. and *T. cruzi*. In addition to enhancing lipophilicity and metabolic stability, some analogs target epigenetic regulators such as HDACs, thereby expanding the parasite‐directed pharmacological profile while potentially influencing host immune pathways. This evolving chemical space highlights the translational promise of piplartine‐based scaffolds as versatile starting points for dual‐acting antiparasitic and host‐directed strategies, rather than fully optimized drug candidates [49, 52].

At the molecular level, the compound’s electrophilic α,β‐unsaturated lactam system confers the ability to covalently modify cysteine residues in redox‐regulatory proteins, leading to oxidative stress in parasites while promoting adaptive antioxidant responses in host cells. This redox bifurcation underlies the dual pharmacology of piplartine, characterized by selective parasite toxicity and modulation of host inflammatory pathways. By activating Nrf2 and suppressing NF‐κB, the compound limits excessive inflammatory signaling and enhances cellular antioxidant defenses. These effects have been validated in macrophages, dendritic cells, and T lymphocytes, where piplartine decreases proinflammatory cytokines such as TNF‐α, IL‐1β, and IL‐6 while promoting IL‐10 production and regulatory T cell polarization. Importantly, these immunomodulatory effects have been described predominantly in noninfectious inflammatory models, including collagen‐induced arthritis, where piplartine attenuates disease severity, and their direct contribution to parasite clearance during infection has not yet been established [29, 53].

The ability of piplartine to selectively inhibit the immunoproteasome without affecting the constitutive proteasome adds an additional dimension to its immunomodulatory profile. This targeted modulation of protein degradation pathways confers anti‐inflammatory effects while minimizing overt cytotoxicity. Nevertheless, immunoproteasome inhibition represents a context‐dependent mechanism, and excessive suppression of antigen processing could theoretically compromise protective immune responses. To date, the impact of piplartine‐mediated immunoproteasome inhibition has not been evaluated in parasite‐infected models. Combined with predicted oral bioavailability, potential BBB permeability, and low acute systemic toxicity in rodents, these attributes support the feasibility of advancing piplartine and selected analogs toward focused preclinical evaluation, rather than immediate translational application.

Pharmacokinetic and drug‐likeness data further support the suitability of piplartine as a preclinical scaffold. In silico analyses indicate high GI absorption, compliance with major drug‐likeness filters (Lipinski, Veber, Egan, and Ghose rules), and the absence of P‐gp substrate behavior, suggesting favorable oral exposure and limited efflux liability. The compound also demonstrates predicted BBB permeability, consistent with activity in neurotropic infection models such as *A. cantonensis*. Experimentally, piplartine exhibits low acute systemic toxicity (LD_50_ > 3000 mg/kg in rodents), reinforcing its preliminary safety profile [9]. However, comprehensive ADME studies and evaluation of long‐term toxicity and immunological safety remain essential prerequisites for translational advancement.

From a translational perspective, the multitarget nature of piplartine represents both an opportunity and a challenge. The compound engages redox‐sensitive signaling, proteasome function, and immune regulation, offering a rare alignment between parasite‐directed activity and host‐directed modulation. Available evidence supports a model in which the antiparasitic effects of piplartine are primarily parasite‐directed, while immunomodulatory actions may function as context‐dependent modifiers of host pathology rather than direct drivers of parasite clearance. This integrated profile may be particularly advantageous in chronic infections characterized by immune‐mediated tissue damage, provided that protective antiparasitic immunity is preserved.

In summary, piplartine exemplifies a natural product with dual biological activity, combining direct antiparasitic efficacy with modulation of host oxidative and inflammatory responses. Its consistent activity across taxonomically diverse pathogens, together with validated immunomodulatory effects in mammalian systems, supports its value as a conceptual and chemical blueprint rather than a finished therapeutic entity. Further preclinical studies integrating pharmacokinetic, toxicological, parasitological, and immunological endpoints will be essential to define its therapeutic window and translational relevance. Together, these findings position piplartine at the interface of natural product chemistry and translational immunoparasitology, highlighting its potential as a platform for the rational development of next‐generation antiparasitic strategies.

**Nomenclature**
BBB: Blood–brain barrier
CC_50_: 50% cytotoxic concentration
cLogP: Calculated logarithm of the partition coefficient
EC_50_: 50% effective concentration
GI: Gastrointestinal
HDAC: Histone deacetylase
HO‐1: Heme oxygenase‐1
IC_50_: 50% inhibitory concentration
IL: Interleukin
IKK: IκB kinase
iNOS: Inducible nitric oxide synthase
LD_50_: 50% lethal dose
L_1_/L_3_: First‐stage/third‐stage larvae
NF‐κB: Nuclear factor kappa‐light‐chain‐enhancer of activated B cells
Nrf2: Nuclear factor erythroid 2–related factor 2
NTDs: Neglected tropical diseases
P‐gp: P‐glycoprotein
PfENR: Plasmodium falciparum enoyl‐acyl carrier protein reductase
PK/PD: Pharmacokinetics/pharmacodynamics
ROS: Reactive oxygen species
SDG: Sustainable development goal
SI: Selectivity index
TNF: Tumor necrosis factor
TPSA: Topological polar surface area
VEGFR: Vascular endothelial growth factor receptor.

## Author Contributions

Lucas Fukui‐Silva and Camila S. Amorim drafted the manuscript. Felipe C. R. de Souza contributed to the discussion and revision of the manuscript. Josué de Moraes designed the study and revised the manuscript.

## Funding

This work was supported by Brazilian funding agencies: the São Paulo Research Foundation (FAPESP) (Grant 2023/08418‐6), the National Council for Scientific and Technological Development (CNPq) (Grants 312211/2021‐0 and 401169/2025‐1), and the Coordination for the Improvement of Higher Education Personnel (CAPES) (Finance Code 001).

## Disclosure

All of the authors have read and approved the final version of the manuscript. The funding agencies had no role in the design of the study, data collection, analysis, interpretation, or in the writing of the manuscript.

## Conflicts of Interest

The authors declare no conflicts of interest.

## Data Availability

The data that support the findings of this study are available from the corresponding author upon reasonable request.
